# Comorbidities and health-related quality of life in Koreans with knee osteoarthritis: Data from the Korean National Health and Nutrition Examination Survey (KNHANES)

**DOI:** 10.1371/journal.pone.0186141

**Published:** 2017-10-18

**Authors:** Hyemin Jeong, Sun Young Baek, Seon Woo Kim, Yeong Hee Eun, In Young Kim, Jaejoon Lee, Chan Hong Jeon, Eun-Mi Koh, Hoon-Suk Cha

**Affiliations:** 1 Division of Rheumatology, Department of Medicine, Soonchunhyang University Hospital, Bucheon, South Korea; 2 Biostatic and Clinical Epidemiology Center, Samsung Medical Center, Seoul, South Korea; 3 Division of Rheumatology, Department of Medicine, Samsung Medical Center, Sungkyunkwan University School of Medicine, Seoul, South Korea; Scientific Institute of Public Health (WIV-ISP), BELGIUM

## Abstract

**Objectives:**

This study aimed to evaluate the association of knee osteoarthritis (OA) with comorbidities and health-related quality of life (HRQOL).

**Methods:**

A total of 8,907 (weighted n = 13,687,058) participants aged ≥50 years who had undergone knee radiography were selected from the 2010–2012 Korea National Health and Nutrition Examination Survey. OA was classified into four subgroups based on the presence or absence of pain and radiographic OA (ROA): non-OA (Pain-/ROA-), pain only (Pain+/ROA-), ROA only (Pain-/ROA+), and painful ROA (Pain+/ROA+). ROA was defined as Kellgren–Lawrence grade ≥ 2. HRQOL measurements including EuroQOL visual analogue scale (EQ-VAS) scores and the five dimensions and summary index of the EuroQOL-5 dimension (EQ-5D index) were also analyzed. Multivariable logistic regression and linear regression analyses were performed.

**Results:**

After adjustment for socioeconomic and lifestyle characteristics, cardiovascular disease, malignancy, and other comorbidities were not significantly associated with OA. Pain only and painful ROA were each significantly associated with limitations in physical activity (odds ratio (OR) 2.66, 95% CI 2.07–3.44, p < 0.001 and OR 2.83, 95% CI 2.25–3.58, p < 0.001, respectively), lower EQ-VAS (β-coefficient = -10.95, p < 0.001 and β-coefficient = -9.75, p < 0.001, respectively), and EQ-5D index (β-coefficient **=** -0.10, p < 0.001 and β-coefficient = -0.13, p < 0.001) compared with the non-OA group, whereas ROA only was not associated with limitations in physical activity or lower HRQOL score.

**Conclusions:**

Comorbidities were not significantly associated with knee OA after adjustment. Knee OA was associated with physical activity and HRQOL. Painful knee OA, with or without ROA, was more strongly associated with decreased physical activity and lower quality of life than ROA without pain.

## Introduction

Osteoarthritis (OA) is the most common musculoskeletal disorder and causes disability in elderly populations [1]. As the population ages, the prevalence of OA increases, and this can cause a considerable economic burden on society [2]. Elderly OA patients who have other emerging comorbidities present a challenge to treating clinicians. OA is characterized by cartilage destruction and subchondral bone remodeling, but the etiology and pathogenesis of OA remain poorly understood. Recent studies reported that proinflammatory cytokines and systemic inflammation are associated with the pathogenesis of OA [3,4]. Rheumatoid arthritis (RA) is a chronic inflammatory disease that is associated with increased prevalence of several comorbidities including cardiovascular disease, infection, malignancy, lung disease, and neuropsychiatric disease [5–8]. In contrast to RA, the precise relationships between OA and these various medical comorbidities remain controversial. In the Framingham Heart Study, symptomatic hand OA was associated with increased risk of coronary heart disease events [9]. In a prospective study, Rahman et al. reported that OA was associated with increased risk of cardiovascular disease [10], and a recent cohort study showed that knee OA was associated with increased risk of all-cause and cardiovascular-specific mortality [11]. Conversely, the Rotterdam study concluded that OA was not associated with increased risk of cardiovascular disease [12]. Several studies on comorbidities other than cardiovascular disease in patients with OA have been published, one of which found that OA was associated with gastritis, obesity, and respiratory infections [13].

OA is the major cause of pain in elderly populations. Arthritic pain causes functional impairment characterized by difficulty with activities of daily living [14]. Depression is associated with reported severity of pain and physical disability in patients with rheumatic disease including OA [15]. Furthermore, the presence of coexistent comorbidities increases the extent of long-term disability in patients with knee OA [16]. Leite et al. reported that OA patients showed a high frequency of metabolic syndrome and depression, which impact both pain and physical function [17]. In addition, higher rates of depression and anxiety cause lower quality of life in patients with OA [18]. Therefore, it is crucial to identify and manage disabilities and pain in patients with OA in routine practice, which will enhance patient quality of life.

However, the data regarding medical comorbidities associated with OA in previous studies were mainly from Western populations. Whether radiographic or symptomatic OA is associated with comorbidities or quality of life remains unclear. The aim of this study was to examine the association of knee OA with comorbidities and health-related quality of life (HRQOL) in a Korean adult population with knee OA compared with those of a corresponding non-OA population after adjustment for socioeconomic and lifestyle characteristics.

## Methods

### Study population

The Korea National Health and Nutrition Examination Survey (KNHANES) is a nationwide survey that is conducted periodically by the Korea Centers for Disease Control and Prevention to investigate the health and nutritional status of the Korean population [19]. This survey assesses the general health and nutrition status of individuals in South Korea through interviews about health and nutrition and basic health assessments. Participants were selected using the proportional allocation-systematic sampling method with multistage stratification to derive a representative Korean population. Although the individual participants are not generally representative of the Korean population, this survey provides representative estimates of the noninstitutionalized Korean civilian population by using the power of sample weight. Every year, 10,000 to 12,000 individuals in approximately 3,800 households are selected from a panel based on the National Census Data. The participation rates of the selected households in the past several cycles of the KNHANES have been high, ranging from 79% to 84%. Among the 25,534 participants in the 2010–2012 KNHANES, 10,152 participants who were 50 years of age or older were selected. Then, 949 participants for whom knee radiograph data or knee pain were missing were excluded. An additional 296 participants who were diagnosed with RA were also excluded. In total, 8,907 participants were selected for analysis. The frequency of missing data for each item was very low, with a maximum frequency of about 1% (0.97%). Written informed consent was obtained from all participants before completing the survey. This study was approved by the institutional review board of Samsung Medical Center (IRB No. 2017-03-041).

### Demographic variables and data collection

The KNHANES was conducted by four special research teams, each composed of eight experts including nurses, nutritionists, and students majoring in public health. The selected professional investigator was placed at the investigation site after completing one month of education and practice. Subsequently, the ability to conduct research was verified through regular education and on-site quality management. A standardized interview was performed in participants’ homes, and an established questionnaire was used to collect information on demographic variables and socioeconomic characteristics. Specifically, data on age, sex, income, region, education, marital status, alcohol consumption, and smoking status were collected. Alcohol consumption was categorized into the following four groups based on the frequency of alcohol consumption during the past year: never, ≤ 1/week, 2-3/week, and ≥ 4/week. Income level was categorized into quartiles based on average individual monthly income. Urban and rural areas were classified by administrative district. Comorbidities were defined in the questionnaire as “Hypertension diagnosed by a physician” through a standardized interview. The question was, “Was your hypertension diagnosed by a physician?” The questionnaire consisted of three responses (1. Yes, 2. No, 3. I have never been sick before). Participants who chose 1 (Yes) were classified as having hypertension. Each interview was conducted individually by a trained professional investigator. Information was also collected on cardiovascular comorbidities including hypertension, dyslipidemia, diabetes, stroke, myocardial infarction, or angina that was diagnosed by a physician. Information was also collected on malignancies including stomach cancer, colon cancer, breast cancer, cervical cancer, lung cancer, and other comorbidities including osteoarthritis, pulmonary tuberculosis, thyroid disease, depression, atopic dermatitis, depression, chronic kidney disease, hepatitis B, hepatitis C, and liver cirrhosis that was diagnosed by a physician. Height and weight were assessed using standardized techniques and equipment. Briefly, height was measured to the nearest 0.1 cm using a portable stadiometer (Seriter, Bismarck, ND, USA). Weight was measured to the nearest 0.1 kg using a Giant-150N calibrated balance-beam scale (Hana, Seoul, Korea). Body mass index (BMI) was calculated by dividing weight by height squared (kg/m^2^).

### Assessment of radiographic OA and knee pain

Radiographic examination of the knee, including the acquisition of bilateral weight-bearing anteroposterior and lateral (30° flexion) plain radiographs, was performed using an SD 3000 Synchro Stand (Accele Ray, Shinyoung Co., Seoul, Korea). Radiographic images were reviewed by two radiologists. The degree of radiographic knee OA was assessed according to the Kellgren-Lawrence (KL) grading system [20]. Radiographs were graded, and concordant grades were accepted by two radiologists. When there was a difference of only one grade between the two radiologists, the higher grade was accepted. If the discrepancy was greater than one grade, a third radiologist was consulted, and the grade concordant with the third grade was accepted. Both sides of the knee were graded, and the higher grade was determined as the KL grade of each participant. The inter-rater agreement between the two radiologists was 95.4%, and the kappa coefficient was 0.74. Participants were classified as having radiographic knee OA if they had a KL grade of 2 or more [21].

Self-reported knee pain (yes/no) was assessed in the standardized interview using the question, “Did you suffer from knee pain for more than 30 days during the last 3 months?” The interview was conducted individually by a trained professional investigator. Knee pain was classified as positive if `yes`was reported.

OA was categorized into four subgroups based on the presence or absence of pain and radiographic OA (ROA) [11]: 1. non-OA (Pain-/ROA-: reference group); 2. Pain only (Pain+/ROA-); 3. ROA only (Pain-/ROA+); and 4. Painful ROA (Pain+/ROA+).

### Quality of life measurement

In the KNHANES, HRQOL is measured using the EuroQOL-5 dimension-3 level (EQ-5D-3L) questionnaire, the EQ-5D index, and the EuroQOL-visual analogue scale (EQ-VAS). These are instruments for measuring HRQOL developed by the EuroQOL group and use of the EQ-5D-3L and EQ-VAS was approved by EuroQOL group (http://www.euroqol.org). The EQ-5D-3L questionnaire consists of five questions for self-reported problems in five dimensions (mobility, self-care, usual activities, pain or discomfort, and depression or anxiety) [22]. There are three levels for each item (1 = no problem, 2 = moderate problem, 3 = severe problem). An EQ-5D validation study confirmed its validity and reliability in the Korean population [23]. The EQ-5D was also validated for measurement of HRQOL in patients with various rheumatic diseases [24] and osteoarthritis [25]. A summary index (EQ-5D index) can be calculated by combining all scores for the five dimensions; the summary index ranges from −0.171 to 1 [26]. The maximum score of 1 on the EQ-5D index indicates the best health, with no problems in any of the five dimensions.

EQ-5Dindex=1−(0.050+0.096×M2+0.418×M3+0.046×SC2+0.136×SC3+0.051×UA2+0.208×UA3+0.037×PD2+0.151×PD3+0.043×AD2+0.158×AD3+0.050×N3)

Substitute a 1 in the formula if the mobility level is 2 or 3 and a 0 if the mobility level is 1. (M2, mobility level 2; M3, mobility level 3; SC2, self-care level 2; SC3, self-care level 3; UA2, usual activities level 2; UA3, usual activities level 3; PD2, pain or discomfort level 2; PD3, pain or discomfort level 3; AD2, anxiety or depression level 2; AD3, anxiety or depression level 3; N3, any dimension level 3).

Participants scored their subjective health status using the EQ-VAS with a vertical scale whose endpoints were labeled `the best health you can imagine`and `the worst health you can imagine`. Scores ranged from 0 (the worst imaginable health state) to 100 (the best imaginable health state).

### Statistical analyses

To reflect representative estimates of the noninstitutionalized Korean civilian population, survey sample weights were applied in all of the analyses. The sample weights were calculated by taking into account the sampling rate, response rate, and age/sex proportions of the reference population (2005 Korean National Census registry). Univariable logistic regression analysis was performed with OA categories as dependent variables and clinical characteristics as independent variables. To analyze the effect of OA on the morbidity and quality of life, multivariable logistic regression models were computed with comorbidities as dependent variables and knee OA categories as independent variables. The models were adjusted for socioeconomic and lifestyle characteristics (age, sex, income, region, marital status, alcohol consumption, smoking status, and BMI). Odds ratios (ORs) with 95% confidence intervals (CIs) of three knee OA categories (pain only, ROA only, painful ROA) were calculated compared with the non-OA category (Pain-/ROA-) for each of the comorbidities. Multivariable linear regression analysis was performed to assess the EQ-VAS score and the EQ-5D index. Overall P-values were provided for the overall effects of clinical characteristics on the OA categories. P–values, ORs, and 95% CIs for the pairwise comparisons between the two OA categories were also presented after correction with Bonferroni`s method for multiple comparisons. Statistical analyses were performed using SAS (SAS version 9.4; SAS Institute, Cary, NC, USA). All *P*-values were two-sided, and a *P* value < 0.05 was considered statistically significant.

## Results

The weighted (n = 13,687,058) demographics and clinical characteristics of the study population are presented in Table 1. Mean participant age was 62.2 years, and 53.2% of the participants were female. The prevalence of college graduates was 11.1%, and mean BMI was 24.0 kg/m^2^. The prevalence of non-OA (Pain-/ROA-), pain only (Pain+/ROA-), ROA only (Pain-/ROA+), and painful ROA (Pain+/ROA+) was 59.4%, 7.9%, 20.7%, and 12.0%, respectively.

**Table 1:** Weighted baseline characteristics of the study population (unweighted, n = 8,907; weighted, n = 13,687,058).

Variables	Weighted mean ± SD or weighted percent
Age, years, mean ± SD	62.2 ± 0.2
Women, %	53.2
Income, %	
Low	26.1
Mid-low	26.0
Mid-high	25.3
High	22.6
Region, %	
Urban	72.5
Rural	27.5
Education, %	
Elementary school	46.0
Middle school	18.7
High school	24.2
College graduation	11.1
Marital status, %	
Married	99.0
Unmarried	1.0
Alcohol consumption, %	
Never	36.3
≤ 1 /week	40.2
2–3 /week	12.8
≥ 4 /week	10.7
Smoking status, %	
Never smoker	55.2
Ex-smoker	25.3
Current smoker	19.5
Body mass index (kg/m^2^), mean ± SD	24.05 ± 0.04
Osteoarthritis, %	
Non-OA (Pain-/ROA-) (reference)	59.4
Pain only (Pain+/ROA-)	7.9
ROA only (Pain-/ROA+)	20.7
Painful ROA (Pain+/ROA+)	12.0
Hypertension, %	37.6
Dyslipidemia, %	16.9
Diabetes, %	13.8
Stroke, %	3.0
Myocardial infarction or angina, %	4.6
Myocardial infarction, %	1.5
Lung cancer, %	0.1
Cervical cancer, %	0.6
Breast cancer, %	0.7
Colon cancer, %	0.7
Stomach cancer, %	1.0
Pulmonary tuberculosis, %	6.4
Asthma, %	3.9
Thyroid disease, %	4.4
Atopic dermatitis, %	1.2
Depression, %	4.8
Chronic kidney disease, %	0.7
Hepatitis B, %	1.6
Hepatitis C, %, %	0.3
Liver cirrhosis, %	0.6

The clinical characteristics analyzed according to knee OA category are shown in Table 2. Overall P-values were given for each variable. Age, sex, income, region, education, alcohol consumption, smoking status, BMI, hypertension, dyslipidemia, diabetes, myocardial infarction or angina, asthma, thyroid disease, and depression all had an effect on OA category. Females exhibited increased odds of OA compared with males and had significantly increased risk of having pain only (OR 2.12, 95% CI 1.37–2.69, p < 0.001), ROA only (OR 2.20, 95% CI 1.86–2.59, p < 0.001), and painful ROA (OR 6.59, 95% CI 5.34–8.14, p < 0.001). Subjects who lived in rural areas showed increased risk of having pain only (OR 1.29, 95% CI 1.02–1.64, p = 0.029), ROA only (OR 1.25, 95% CI 1.04–1.50, p = 0.012), and painful OA (OR 1.87, 95% CI 1.50–2.33, p < 0.001) compared with subjects who lived in urban areas. Smoking and alcohol consumption were less common in subjects with OA than in the non-OA group. Current smokers were less likely to have pain only (OR 0.55, 95% CI 0.39–0.79, p < 0.001), ROA only (OR 0.40, 95% CI 0.31–0.53, p < 0.001), and painful OA (OR 0.24, 95% CI 0.17–0.35, p < 0.001) compared with never smokers. Subjects who consumed alcohol more than four days a week were less likely to have pain only (OR 0.48, 95% CI 0.29–0.79, p = 0.004), ROA only (OR 0.64, 95% CI 0.46–0.89, p = 0.002), and painful ROA (OR 0.23, 95% CI 0.15–0.37, p < 0.001) compared with never drinkers. The prevalence of cardiovascular risk factors and cardiovascular disease, including hypertension, dyslipidemia, diabetes, and myocardial infarction or angina, was higher in participants with OA compared with non-OA participants. Subjects with hypertension (OR 2.64, 95% CI 2.16–3.22, p < 0.001), dyslipidemia (OR 1.33, 95% CI 1.01–1.68, p = 0.014), and diabetes (OR 1.45, 95% CI 1.12–1.88, p = 0.002) showed increased risk of painful ROA. There was no significant association between solid malignancy and OA category. Subjects with asthma (OR 2.34, 95% CI 1.54–3.58, p < 0.001) and thyroid disease (OR 1.55, 95% CI 1.04–2.29, p = 0.025) were more likely to have painful ROA than non-OA. Depression was associated with increased risk of pain only (OR 2.07, 95% CI 1.37–3.12, p < 0.001).

**Table 2:** Clinical characteristics and comorbidities across knee status categories.

	Non-OA (Pain-/ROA-) (Weighted n = 8,131,884)	Pain only (Pain+/ROA-) (Weighted n = 1,093,947)	ROA only (Pain-/ROA+) (Weighted n = 2,819,587)	Painful ROA (Pain+/ROA+) (Weighted n = 1,641,640)	P value
Age, years, mean ± SD	59.4 ± 0.2	62.1 ± 0.4	66.1 ± 0.3	69.4 ± 0.4	< 0.001
Female, %	42.9	61.4	62.2	83.2	< 0.001
Income, %					< 0.001
Low	24.8	30.1	24.7	32.0	
Mid-low	24.8	29.5	27.2	27.5	
Mid-high	26.5	21.5	24.3	23.7	
High	23.9	18.9	23.8	16.8	
Region, %					< 0.001
Urban	76.4	69.7	69.4	60.5	
Rural	23.6	30.3	30.6	39.5	
Education, %					< 0.001
Elementary school	34.6	56.2	54.9	80.0	
Middle school	20.5	19.4	17.4	11.6	
High school	30.1	17.4	20.0	6.9	
College graduation	14.8	7.0	7.7	1.5	
Marital status, %					0.066
Married	98.8	98.2	99.4	99.6	
Unmarried	1.2	1.8	0.6	0.4	
Alcohol consumption, %					< 0.001
Never	30.1	42.0	42.4	53.2	
≤ 1 /week	42.7	37.9	37.2	34.8	
2–3 /week	15.1	12.1	9.5	7.0	
≥ 4 /week	12.1	8.0	10.9	5.0	
Smoking status, %					< 0.001
Never smoker	46.8	61.1	63.8	78.1	
Ex-smoker	29.3.	21.6	23.0	12.2	
Current smoker	23.9	17.3	13.2	9.7	
Body mass index (kg/m^2^), mean ± SD	23.6 ± 0.1	23.9 ± 0.2	24.6 ± 0.1	25.2 ± 0.1	< 0.001
Hypertension, %	31.2	35.5	46.8	54.5	< 0.001
Dyslipidemia, %	15.6	20.1	17.8	19.7	0.006
Diabetes, %	12.7	13.2	15.1	17.4	0.003
Stroke, %	2.7	3.0	3.5	4.0	0.177
Myocardial infarction or angina, %	4.0	5.7	5.4	5.2	0.038
Myocardial infarction, %	1.3	2.2	1.7	1.5	0.536
Angina, %	2.9	3.9	4.0	4.0	0.081
Lung cancer, %	0.1	0.1	0.2	0.1	0.309
Cervical cancer, %	0.4	1.0	0.8	1.0	0.121
Breast cancer, %	0.6	1.0	0.5	1.1	0.179
Colon cancer, %	0.7	0.5	0.5	0.8	0.662
Stomach cancer, %	1.1	1.2	1.0	0.6	0.428
Pulmonary tuberculosis, %	6.9	5.9	5.6	5.4	0.218
Asthma, %	3.0	6.0	4.0	6.8	< 0.001
Thyroid disease, %	3.7	7.3	4.6	5.6	0.001
Atopic dermatitis, %	1.0	2.3	1.4	0.8	0.062
Depression, %	4.4	8.7	3.5	6.5	< 0.001
Chronic kidney disease, %	0.5	1.2	0.8	1.0	0.075
Hepatitis B, %	1.9	1.4	1.0	1.3	0.087
Hepatitis C, %	0.3	0.4	0.3	0.2	0.754
Liver cirrhosis, %	0.5	0.5	1.0	0.3	0.225

Overall P-values were provided for the overall effects of clinical characteristics on the OA categories.

The physical activity and HRQOL measures, including the weighted proportions of problems of each of the EQ-5 dimensions, the EQ-VAS scores, and the EQ-5D indices, are presented in Table 3. Activity limitation, cause of activity limitation, mobility, self-care, usual activities, pain/discomfort, anxiety/depression, EQ-VAS, and EQ-5D index were significantly associated with OA category. Participants with limited physical activity were more likely to have pain only (OR 3.27, 95% CI 2.43–4.40, p < 0.001) and painful ROA (OR 4.70, 95% CI 3.68–6.00, p < 0.001) compared to those with no physical limitations. Limited physical activity was not significantly associated with ROA only (OR 1.23, 95% CI 0.98–1.54, p = 0.097). Participants who had severe problems with mobility (OR 30.42, 95% CI 13.58–68.14, p < 0.001), self-care (OR 12.20, 95% CI 4.44–33.51, p < 0.001), usual activities (OR 17.76, 95% CI 9.55–33.02, p < 0.001), pain/discomfort (OR 25.49, 95% CI 16.02–40.56, p < 0.001), and anxiety/depression (OR 7.13, 95% CI 3.23–15.75, p < 0.001) were more likely to have painful ROA compared with participants with no such problems. Participants who had severe problems with mobility (OR 9.84, 95% CI 3.75–25.83, p < 0.001), self-care (OR 7.08, 95% CI 1.87–26.80, p < 0.001), usual activities (OR 8.30, 95% CI 4.00–17.23, p < 0.001), pain/discomfort (OR 12.11, 95% CI 7.11–20.62. p < 0.001), and anxiety/depression (OR 8.36, 95% CI 3.71–18.82, p < 0.001) were more likely to have pain only compared with participants with no such problems. There was no association with ROA only and severe problems with mobility (OR 1.15, 95% CI 0.39–3.38, p = 1.000), self-care (OR 1.19, 95% CI 0.26–5.41, p = 1.000), pain/discomfort (OR 1.37, 95% CI 0.80–2.34, p = 0.710), or anxiety/depression (OR 0.98, 95% CI 0.31–3.07, p = 1.000), while there was a significant association with usual activities (OR 2.89, 95% CI 1.40–5.98, p = 0.001). Increased EQ-VAS score was associated with decreased risk of pain only (OR 0.97, 95% CI 0.97–0.98, p < 0.001) and painful ROA (OR 0.97, 95% CI 0.96–0.97, p <0.001). EQ-VAS score was not significantly associated with ROA only (OR 0.99, 95% CI 0.99–1.00, p = 0.055). Increased EQ-5D index showed significantly lower risk of pain only, ROA only, and painful ROA than non-OA (OR 0.001, 95% CI 0.000–0.003, p < 0.001, OR 0.141, 95% CI 0.059–0.336, p < 0.001, and OR 0.001, 95% CI 0.000–0.001, p < 0.001, respectively).

**Table 3:** Physical activity and health-related quality of life measures across knee status categories.

	Non-OA (Pain-/ROA-) (Weighted n = 8,131,884)	Pain only (Pain+/ROA-) (Weighted n = 1,093,947)	ROA only (Pain-/ROA+) (Weighted n = 2,819,587)	Painful ROA (Pain+/ROA+) (Weighted n = 1,641,640)	P value
Activity limitation					< 0.001
Yes, %	11.7	28.9	13.3	36.9	
No, %	88.9	71.1	86.7	63.1	
Cause of activity limitation					< 0.001
Arthralgia	0.6	9.6	1.5	22.7	
Other	99.4	90.4	98.5	77.3	
Mobility					< 0.001
I have no problems walking	86.1	51.4	74.1	26.3	
I have moderate problems walking	13.4	45.5	25.4	68.7	
I am unable to walk	0.5	3.1	0.5	5.0	
Self-care					< 0.001
I have no problems washing or dressing myself	95.1	85.1	94.6	77.4	
I have moderate problems washing or dressing myself	4.7	13.6	5.2	20.6	
I am unable to wash or dress myself	0.2	1.3	0.2	2.0	
Usual activities					< 0.001
I have no problems doing my usual activities	91.1	66.1	98.6	53.3	
I have moderate problems doing my usual activities	8.2	29.8	10.5	39.6	
I am unable to do my usual activities	0.7	4.1	1.9	7.1	
Pain/discomfort					< 0.001
I have no pain or discomfort	78.2	41.9	77.4	30.9	
I have moderate pain or discomfort	20.1	47.2	20.3	52.1	
I have extreme pain or discomfort	1.7	10.9	2.3	17.0	
Anxiety/ depression					< 0.001
I am not anxious or depressed	88.4	70.2	89.6	74.5	
I am moderately anxious or depressed	11.1	26.1	9.9	22.1	
I am extremely anxious or depressed	0.6	3.7	0.5	3.4	
EQ-VAS score	73.72 ± 0.35	60.99 ± 1.06	72.21 ± 0.56	59.70 ± 0.92	< 0.001
EQ-5D index	0.94 ± 0.00	0.82 ± 0.01	0.92 ± 0.00	0.76 ± 0.01	< 0.001

Overall P-values were provided for the overall effects of clinical characteristics on the OA categories.

Table 4 shows the adjusted ORs for medical comorbidities by knee OA category compared with non-OA. After adjusting for socioeconomic and lifestyle characteristics (age, sex, income, region, education, marriage, alcohol consumption, smoking status, and BMI), OA was not associated with cardiovascular disease. The prevalence of colon cancer was significantly decreased in subjects with ROA only compared with non-OA subjects. The pain only group was associated with increased prevalence of asthma, thyroid disease, depression, and atopic dermatitis compared with the non-OA group. The prevalence of physical activity limitations was significantly increased in the pain only and painful ROA groups compared with the non-OA group.

**Table 4:** Adjusted ORs (95% CIs) for medical comorbidities by knee OA category compared with the non-OA (Pain-/ROA-) group.

	OR (95% CI) Pain only (Pain+/ROA-) vs. Non-OA (Pain-/ROA-)	P value	OR (95% CI) ROA only (Pain-/ROA+) vs. Non-OA (Pain-/ROA-)	P value	OR (95% CI) Painful ROA (Pain+/ROA+) vs. Non-OA (Pain-/ROA-)	P value
Hypertension	0.97 (0.79–1.21)	0.798	1.09 (0.95–1.26)	0.230	1.12 (0.94–1.33)	0.207
Dyslipidemia	1.29 (1.01–1.66)	0.045	1.00 (0.82–1.22)	0.994	1.02 (0.80–1.30)	0.872
Diabetes	0.96 (0.71–1.30)	0.800	0.95 (0.77–1.18)	0.631	1.03 (0.81–1.29)	0.834
Stroke	0.87 (0.53–1.42)	0.566	0.92 (0.64–1.31)	0.635	0.97 (0.62–1.52)	0.891
Myocardial infarction or angina	1.38 (0.89–2.14)	0.152	0.99 (0.74–1.33)	0.946	0.92 (0.62–1.37)	0.689
Myocardial infarction	1.84 (0.89–3.82)	0.100	0.95 (0.55–1.63)	0.841	1.12 (0.54–2.35)	0.756
Angina	1.19 (0.70–2.00)	0.520	0.99 (0.71–1.39)	0.969	0.85 (0.56–1.30)	0.454
Lung cancer	0.34 (0.04–2.99)	0.332	2.09 (0.60–7.19)	0.244	0.79 (0.17–3.75)	0.770
Cervical cancer	1.96 (0.62–6.20)	0.252	1.44 (0.57–3.68)	0.444	1.32 (0.41–4.29)	0.645
Breast cancer	1.61 (0.68–3.79)	0.276	0.76 (0.29–1.99)	0.574	1.42 (0.54–3.71)	0.478
Colon cancer	0.57 (0.20–1.63)	0.291	0.28 (0.11–0.72)	0.009	0.60 (0.25–1.41)	0.239
Stomach cancer	1.24 (0.61–2.53)	0.558	0.90 (0.44–1.85)	0.767	0.59 (0.22–1.55)	0.280
Pulmonary tuberculosis	0.97 (0.64–1.48)	0.886	1.02 (0.75–1.38)	0.917	1.21 (0.81–1.81)	0.348
Asthma	1.55 (1.01–2.39)	0.048	1.03 (0.69–1.54)	0.884	1.47 (0.95–2.28)	0.081
Thyroid disease	1.88 (1.25–2.83)	0.002	1.17 (0.86–1.59)	0.331	1.36 (0.93–2.01)	0.117
Depression	1.73 (1.22–2.45)	0.002	0.74 (0.51–1.07)	0.111	1.11 (0.76–1.61)	0.588
Atopic dermatitis	2.53 (1.26–5.08)	0.009	1.73 (0.77–3.87)	0.181	1.21 (0.48–3.06)	0.686
Chronic kidney disease	2.57 (0.92–7.18)	0.071	1.67 (0.68–4.10)	0.262	1.87 (0.63–5.51)	0.257
Hepatitis B	1.00 (0.48–2.08)	0.994	0.75 (0.40–1.41)	0.364	1.75 (0.66–4.65)	0.260
Hepatitis C	1.16 (0.32–4.15)	0.825	0.70 (0.31–1.61)	0.402	0.43 (0.06–2.89)	0.383
Liver cirrhosis	1.06 (0.32–3.56)	0.926	1.80 (0.88–3.66)	0.107	0.63 (0.19–2.03)	0.436
Physical activity limitation	2.66 (2.07–3.44)	< 0.001	0.91 (0.75–1.12)	0.384	2.83 (2.25–3.58)	< 0.001

Adjusted for age, sex, income, region, education, marital status, alcohol consumption, smoking status, and body mass index

Compared with the non-OA group, the pain only and painful ROA groups were significantly associated with decreased EQ-VAS score and decreased EQ-5D index after adjustment for socioeconomic and lifestyle characteristics (Table 5).

**Table 5:** Multivariable linear regression model for EuroQOL score and EQ-5D index by knee OA category compared with the non-OA (Pain-/ROA-) group.

	β-coefficient ± SE Pain only (Pain+/ROA-) vs. Non-OA (Pain-/ROA-)	P value	β-coefficient ± SE ROA only (Pain-/ROA+) vs. Non-OA (Pain-/ROA-)	P value	β-coefficient ± SE Painful ROA (Pain+/ROA+) vs. Non-OA (Pain-/ROA-)	P value
EQ-VAS	-10.95 ± 1.09	< 0.001	0.51 ± 0.67	0.448	-9.75 ± 0.98	< 0.001
EQ-5D index	-0.10 ± 0.01	< 0.001	0.01 ± 0.00	0.005	-0.13 ± 0.01	< 0.001

Adjusted for age, sex, income, region, education, marital status, alcohol consumption, smoking status, and body mass index.

## Discussion

We investigated the comorbidities and HRQOL of patients with knee OA using data from a nationwide survey conducted by the Korean government. We found that cardiovascular disease was not significantly associated with OA. These findings corresponded well with those of a previous study reporting that OA is not a significant predictor of incident cardiovascular disease in elderly Japanese-American males [27]. However, the findings of the present study contrast with the results of the US National Health and Nutrition Examination Survey [28]. The US survey reported that US adults with OA have a high prevalence of cardiovascular risk factors compared with an age- and sex-matched general population without arthritis. In the Canadian Community Health Survey, OA was associated with increased prevalence of cardiovascular disease [29]. Although a recent meta-analysis showed that OA is a significant risk factor for cardiovascular disease [30], this topic remains controversial. In contrast to OA, the incidence of cardiovascular disease is higher among patients with RA [31]. The increased risk of cardiovascular disease observed in patients with RA is comparable to the risk in patients with type 2 diabetes mellitus [32,33]. Autoimmune diseases such as RA and systemic lupus erythematosus increase the risk of cardiovascular morbidity and mortality. Rheumatoid joints and atherosclerotic lesions share common proinflammatory features [34]. Moreover, systemic inflammation in autoimmune disease causes endothelial dysfunction and accelerates atherosclerosis; failure to control this inflammation leads to atherosclerosis [35–37]. OA is classified as a non-inflammatory type of arthritis. However, evidence of low-grade systemic inflammation in OA exists [38,39]. Synovitis is present in a significant proportion of patients with primary OA, and inflammation has been implicated in the pathogenesis of OA [40]. Nielen et al. reported that the prevalence of cardiovascular disease is increased to similar levels in patients with inflammatory arthritis and diabetes mellitus, while OA is not associated with cardiovascular disease [41]. Systemic inflammatory load is critical to the development of cardiovascular disease. The findings of the current study suggest that systemic inflammation in OA is insufficient to drive the development of cardiovascular disease.

However, many published studies have reported that musculoskeletal disease causes metabolic syndrome. Metabolic syndrome components include central obesity, hypertension, impaired fasting glucose, and dyslipidemia. The presence of metabolic syndrome is significantly associated with the risk of knee OA [42,43]. The Japanese Research on Osteoarthritis Against Disability (ROAD) study also reported that knee OA is associated with components of metabolic syndrome, and that the number of metabolic syndrome components is significantly associated with the presence of knee OA [44]. Metabolic syndrome was a potential risk factor for disability in the elderly US population in the National Health and Nutrition Examination Survey (NHANES) [45]. In the Framingham osteoarthritis study, metabolic syndrome was associated with the incidence of both radiographic OA and symptomatic OA, but these associations were no longer significant after BMI adjustment [46]. Overweight is a well-known risk factor for knee OA due to abnormal knee joint loading [47]. Also, overweight (especially central obesity) is associated with increased release of adipokines by abdominal adipose tissue, which results in low-grade inflammatory status in many peripheral tissues, including joint tissue [48]. In the Michigan Study of Women's Health Across the Nation, serum leptin level was associated with knee OA [49]. Moreover, adipose tissue in the infrapatellar fat pads has been considered a potential source of adipokines [50]. Inflammation, including synovitis and effusion, is associated with responsiveness of the peripheral nociceptive neurons, resulting in pain sensitization in knee OA [51,52]. Pain in knee OA caused by synovial inflammation is related to decreased morbidity, which leads to obesity. Increased systemic inflammation caused by adipokines leads to metabolic syndrome [53]. Metabolic syndrome is associated with increased risk of cardiovascular disease [54,55], and both metabolic syndrome and increased BMI are risk factors for knee OA. Multiple factors are related to incidence of knee OA, and the interactions between metabolic syndrome, cardiovascular disease, and OA remain unresolved. In the current study, we did not identify an association between knee OA and cardiovascular disease. Also, metabolic syndrome has not been reported to be significantly associated with knee OA in the Korean population [56]. BMI is lower in Asian populations than in Western populations [57]. This suggests that factors other than body weight might be more highly associated with the pathogenesis of knee OA in Korean patients and that lower adipokine level might result in lower cardiovascular disease risk in the Korean population. However, we could not determine causality due to the cross-sectional nature of this study. Further investigation is needed regarding the causality of the relationships between knee OA and cardiovascular comorbidities.

Chronic inflammation is also associated with cancer risk. In the current study, malignancy was not significantly associated with OA, which is similar to the results obtained in a Western study [13]. Subjects in the ROA only group in our study were at lower risk of colon cancer. Nonsteroidal anti-inflammatory drugs (NSAIDs) decrease the risk of colorectal cancer by cyclooxygenase (COX) regulation [58]. Using NSAIDs in the management of OA might result in a low prevalence of colon cancer in patients with OA. However, we have no data on the use of NSAIDs in subjects with OA and thus could not draw conclusions between cancer and OA in the current study. Moreover, only the ROA only group showed a statistical significant relationship between OA and colon cancer; thus, it is difficult to reach any definitive conclusions. Inflammatory cytokines such as IL-6, CRP, and TNF-alpha have been shown to be associated with cancer incidence [59]. Considering that chronic inflammation is also associated with the risk of cancer and the findings of the current study, we suggest that OA is not associated with increased risk of malignancy. In the pain only group, the prevalence of asthma, thyroid disease, depression, and atopic dermatitis was significantly higher than that in the non-OA group. A Western study showed that the prevalence of endocrine disorders, mental disorders, respiratory system diseases, and skin and subcutaneous tissue diseases was elevated in subjects with OA compared with control subjects [13]. Patients with OA had several comorbidities, including cardiovascular disease, diseases of eye, ear, nose, throat, urogenital disease, and endocrine disease. The number and severity of morbidities were associated with limited physical activity and pain [60]. In the current study, many of these comorbidities including asthma, thyroid disease, and atopic dermatitis were significantly different only in the pain only group. Although pain is associated with systemic inflammation leading to an increased incidence of medical comorbidities, comorbidities were not significantly associated with painful ROA. Thus, our data do not directly support an association between OA and medical comorbidity.

We found that HRQOL indices, including EQ-VAS score and EQ-5D index, were significantly lower in subjects with pain only and in subjects with painful ROA. This finding is consistent with those of a previous cross-sectional study, which reported that patients with symptomatic knee OA had significantly lower QOL than patients with radiographic knee OA without pain [61]. Musculoskeletal diseases are the most common cause of pain worldwide, and pain is the primary concern of patients with OA [62]. Pain is the main cause of total knee replacement surgery in patients with OA. However, a few patients experience continued unexplained pain after knee replacement surgery [63]. Many studies have reported that radiological changes in the knee joint are only weakly related to joint pain [64,65]. Various mechanisms are involved in the pain pathogenesis of OA. Changes in the nociceptive process caused by local inflammation of subchondral bone and the synovium as well as peripheral neuronal sensitization contribute to OA pain. Also, central pain sensitization is important in the pathophysiology of pain in OA [66,67]. Therefore, a neuropathic pain component can be a predominant feature in individuals with minor joint changes and high levels of pain. In these patients, centrally oriented medications are recommended rather than analgesic treatment that is involved in peripheral processes. Central pain sensitization is related to psychosocial factors [68]. Psychosocial factors are also key determinants of pain severity [64]. Individuals with OA who require treatment for pain are more likely to have depressive symptoms [69]. This suggests that the lower quality of life observed in subjects with knee pain regardless of the presence of radiographic changes might be related to a central pain sensitization pathway.

Knee OA can lead to considerable functional limitations associated with pain. Limitations in activities of daily living and recreation in patients with OA affect HRQOL as well as pain. Subjects with musculoskeletal disease showed significantly lower HRQOL than those without musculoskeletal disease, and patients with OA reported worse HRQOL patterns compared with patients with other chronic disease [70]. In Japanese elderly women with knee OA, quality of life was associated with physical activity [71]. As OA progresses, HRQOL progressively declines [72]. Progression of knee OA decreases physical activity and activities of daily living and therefore affects mental health and quality of life. Functional independence is associated with higher QOL in patients with OA [73]. Considering the impact of OA on HRQOL, clinicians need to assess and manage both pain and physical activity limitations.

The present study has some limitations. First, comorbidity diagnoses depended on the information self-reported by the participants in an interview. This is a major limitation of this type of nationwide survey because patients might misunderstand the information given by physicians. Although trained researchers asked the study population about diagnoses during face-to-face interviews, the risk of recall bias should be considered. Second, we could not assess medications for OA and were unable to evaluate the potential associations between comorbidities and medications. Third, although we defined OA based on the presence of pain and knee radiographs, no classification criteria were applied for diagnosis of OA. Subjects in the pain only group were defined as having a KL grade < 2 OA with knee pain for 30 days or longer within the past 3 months. Although we excluded subjects with RA, other knee joint pathologies such as trauma could not be excluded. However, considering the prevalence of arthritis in the elderly population [74] and symptom duration, most participants in the pain only group likely had knee OA. Fourth, we could not determine causality with our cross-sectional study design. Fifth, BMI varies by race/ethnicity and sex and does not provide information about muscularity; thus, BMI is not an ideal surrogate marker for joint loading. Adjustment for body composition rather than BMI is more useful in knee OA studies. Finally, we could not use the Western Ontario and MacMaster Universities Osteoarthritis Index (WOMAC) in the assessment of OA. The WOMAC provides detailed information about symptoms of OA including pain, stiffness, and physical function. If WOMAC data had been available in this study, it would have provided more evidence regarding the impact of OA on HRQOL. In addition, KNHANES data provide information on EQ-5D-3L as a measure of quality of life. The EQ-5D-5L is a more recent and updated form of this measure that provides a wider range of information than the EQ-5D-3L. The strength of our study is that we analyzed a large, nationally representative dataset of the Korean elderly population that had undergone knee radiography. Moreover, we analyzed four subgroups to evaluate whether symptomatic or radiographic OA is associated with comorbidities or HRQOL.

In conclusion, we did not identify any association between knee OA and comorbidities after adjustment for various factors. Knee OA was associated with limited physical activity and poor QOL. Painful OA with or without ROA was more significantly associated with a decline in physical activity and lower quality of life than was ROA without pain. Since the incidence of OA in the elderly is rising concomitantly with life expectancy, clinicians should focus on QOL in their daily clinical practice. Our study suggests that interventions to relieve knee pain could be effective for improving HRQOL in patients with OA.

## Supporting information

S1 File Raw data used for this study. (XLSX)
